# Surfactant Protein D Inhibits HIV-1 Infection of Target Cells via Interference with gp120-CD4 Interaction and Modulates Pro-Inflammatory Cytokine Production

**DOI:** 10.1371/journal.pone.0102395

**Published:** 2014-07-18

**Authors:** Hrishikesh Pandit, Sandhya Gopal, Archana Sonawani, Ajit Kumar Yadav, Asif S. Qaseem, Himangi Warke, Anushree Patil, Rahul Gajbhiye, Vijay Kulkarni, Maha Ahmed Al-Mozaini, Susan Idicula-Thomas, Uday Kishore, Taruna Madan

**Affiliations:** 1 Department of Innate Immunity, National Institute for Research in Reproductive Health (ICMR), Mumbai, Maharashtra, India; 2 Biomedical Informatics Centre, National Institute for Research in Reproductive Health (ICMR), Mumbai, Maharashtra, India; 3 Centre for Infection, Immunity and Disease Mechanisms, Brunel University, London, United Kingdom; 4 Department of Obstetrics and Gynecology, Seth G S Medical College and K E M Hospital, Mumbai, Maharashtra, India; 5 Department of Clinical Research, National Institute for Research in Reproductive Health (ICMR), Mumbai, Maharashtra, India; 6 Immunocompromised Host Research Section, Department of Infection and Immunity, King Faisal Specialist Hospital and Research Centre, Riyadh, Saudi Arabia; Boston University School of Medicine, United States of America

## Abstract

Surfactant Protein SP-D, a member of the collectin family, is a pattern recognition protein, secreted by mucosal epithelial cells and has an important role in innate immunity against various pathogens. In this study, we confirm that native human SP-D and a recombinant fragment of human SP-D (rhSP-D) bind to gp120 of HIV-1 and significantly inhibit viral replication *in vitro* in a calcium and dose-dependent manner. We show, for the first time, that SP-D and rhSP-D act as potent inhibitors of HIV-1 entry in to target cells and block the interaction between CD4 and gp120 in a dose-dependent manner. The rhSP-D-mediated inhibition of viral replication was examined using three clinical isolates of HIV-1 and three target cells: Jurkat T cells, U937 monocytic cells and PBMCs. HIV-1 induced cytokine storm in the three target cells was significantly suppressed by rhSP-D. Phosphorylation of key kinases p38, Erk1/2 and AKT, which contribute to HIV-1 induced immune activation, was significantly reduced *in vitro* in the presence of rhSP-D. Notably, anti-HIV-1 activity of rhSP-D was retained in the presence of biological fluids such as cervico-vaginal lavage and seminal plasma. Our study illustrates the multi-faceted role of human SP-D against HIV-1 and potential of rhSP-D for immunotherapy to inhibit viral entry and immune activation in acute HIV infection.

## Introduction

Acute HIV infection is marked by a pro-inflammatory “cytokine storm” that promotes viral replication and mediate immunopathology [1]. This leads to a non-specific activation and proliferation of naïve CD4^+^ T cells providing the ideal micro-environment for viral replication. Resident macrophages and CD4^+^ T cells in the draining lymph nodes are one of the first immune cells that come in contact with the virus. Upon viral challenge, monocytes/macrophages induce high levels Th1 cytokines (IFN-γ, IL-2 and IL-12), pro-inflammatory cytokines (TNF-α, IL-1β, IL-6) and certain chemokines that favor the formation of viral reservoirs with strongly increased viral transcription [2]. Similarly, infected CD4^+^ T cells are known to induce levels of IL-2, IL-6, and TNF-α and synergistically induce HIV-1 replication [3]. These series of events lead to successful viral entry and dissemination. Thus, a protective anti-viral response would require a tight regulation of the excessive immune activation. Recent anti-HIV-1 vaccine design and prevention strategies are focused on regulating such initial, generalized immune activation that can curb viral replication [4].

Surfactant Protein SP-D, a member of the collectin family, is a pattern recognition innate immune molecule that brings about clearance of various pathogens via agglutination, and enhanced phagocytosis and killing. In the process, SP-D also modulates pathogen mediated pro-inflammatory effects on macrophages and T cells [5]. The primary structure of human SP-D is characterized by an N-terminal, triple-helical collagen region, an α-helical coiled-coil neck region, and a homotrimeric C-type lectin domain or carbohydrate recognition domain (CRD), which resembles Surfactant Protein SP-A (SP-A) and Mannose Binding Lectin (MBL), other members of the collectin family [6]. The trimeric CRD interacts with glycosylated moieties on the pathogen surface, mediating agglutination and inhibiting infectivity. Through its CRD and collagen domains, SP-D interacts with immune cells via various receptors such as calreticulin/CD91, SIRPα, and CD14 on the cell surface, and thus, regulates effector functions [7], [8]. SP-D down-regulates LPS-elicited inflammatory responses by macrophage and inhibits TNF-α production [9]. SP-D also exerts its inhibitory effects on the proliferation of CD4^+^ T cells and IL-2 production [10], [11]. In response to allergens, SP-D modulates T cells response by up-regulating CTLA4; a negative regulator of T cell activation [12]. Similar to SP-D, a recombinant fragment of SP-D, composed of homotrimeric neck and CRD region, has been shown to reduce inflammation in several pathophysiological conditions [13].

SP-D binds to the HIV-1 surface protein gp120 and significantly inhibits replication in U937 monocytes [14] and PM1 T cells [15]. Moreover, SP-D is secreted by mucosal epithelial cells and is present in the vaginal tract [16], and thus, likely to be relevant for sexual transmission of HIV. There are reports that appear to suggest regulation of SP-D levels in different stages of HIV infection. Serum SP-D levels are increased in patients with AIDS, but not in early HIV infection [17]. On initiation of anti-retroviral therapy (ART) and subsequent suppression of HIV replication, serum SP-D levels are reported to decrease significantly [18]. These two clinical correlates suggest regulation of SP-D expression during HIV pathogenesis.

Using three clinical isolates of HIV-1 that are different in tropism, and three target cells, we show here that SP-D as well as rhSP-D are potent inhibitors of HIV entry and block the interaction between CD4 and gp120 in a dose-dependent manner. The rhSP-D also suppresses significantly pro-inflammatory cytokines induced by HIV-1 in Jurkat T cells, U937 monocytes and activated PBMCs. Such cytokine suppression appears to be associated with reduced signaling as evident from reduced phosphorylation of AKT and MEK/ERK in presence of rhSP-D. Thus, SP-D has a multi-faceted and hierarchical protective role in host defense against HIV-1. We also report that the anti-HIV-1 properties of rhSP-D remain unaltered in the presence of cervico-vaginal lavage and seminal plasma, raising the possibility that the recombinant protein may have a prophylactic value.

## Results

### SP-D and rhSP-D recognize HIV-1 glycoproteins gp41, gp120 and gp160

**Figure 1:**
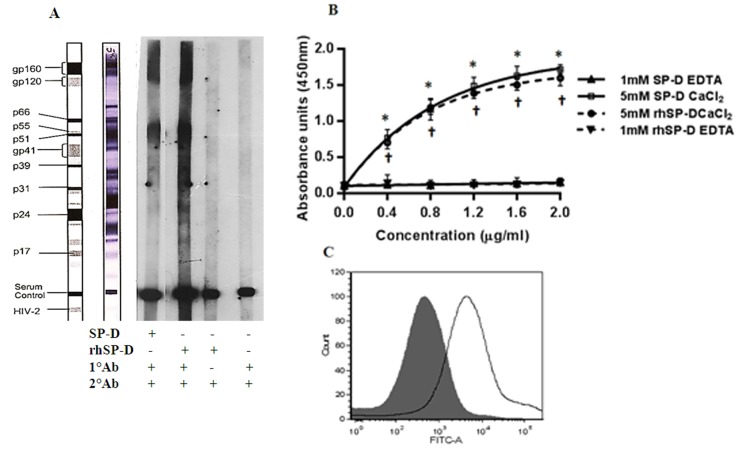
SP-D and rhSP-D recognize HIV-1 gp41, gp120 and gp160. (**A**) A representative image of a ligand blot analysis (n = 3) using a ready-to-use HIV-1 antigen PVDF strip, probed with SP-D or rhSP-D (1 µg/ml) in presence of 2 mM CaCl_2_ and developed using a monoclonal antibody against human SP-D and a 1∶1000 anti-mouse IgG-peroxidase. SP-D and rhSP-D specifically bind to HIV-1 glycoproteins gp41, gp120 and gp 160. (**B**) ELISA assay showing the binding of different concentrations of SP-D (solid line) and rhSP-D (dotted line) to immobilized recombinant r-gp120 (2 µg/mL). SP-D or rhSP-D bind to gp120 in the presence of 5 mM CaCl_2_, and the interaction is inhibited by 1 mM EDTA. Each data point represents the mean ± S.D. (n = 4). †, * shows a statistically significant increase in the binding of SP-D or rhSP-D respectively to HIV-1 gp120 in the presence of calcium compared to EDTA (p<0.05) (**C**) A representative histogram of FITC labeled gp120 on HL2/3 cells (n = 3). HIV-1 gp120 expressing HL2/3 cells were incubated with rhSP-D (2 µg/mL) or no rhSP-D and further probed with a polyclonal FITC-tagged anti-gp120 antibody. In presence of rhSP-D, significantly lesser FITC signal was detected by flow cytometry (gray tinted histogram) comparing to no rhSP-D (black lined histogram).

SP-D has previously been shown to bind gp120 [14], [15]. We carried out ligand blot analysis in order to assess what HIV-1 antigens were being recognized by SP-D and rhSP-D using a commercially available HIV antigen coated strip. Both SP-D and rhSP-D showed binding to envelope HIV-1 glycoproteins gp41, gp120 and gp160 (Fig 1A). No binding was observed to any other HIV-1 antigens. When soluble HIV-1 gp120 was immobilized and probed with serial dilutions of SP-D or rhSP-D (0.4–2 µg/ml), there was a dose- and calcium-dependent binding that was inhibited by EDTA (Fig 1B). Since structural conformation of surface expressed gp120 may impose restrictions on the flexibility of this interaction, we evaluated the interaction of gp120 expressing HL2/3 cells with SP-D using a FITC labeled polyclonal antibody raised against gp120. The mean fluorescence intensity of the FITC labeled cells was reduced significantly in presence of rhSP-D (Fig 1C) as well as SP-D (data not shown) compared to untreated control.

### *In silico* analysis of SP-D -gp120 interaction

*In silico* studies were carried out to identify potential binding sites of SP-D in gp120. Since, experimental observations have shown that SP-D binds gp120 through glycan mediated interactions [14], we screened the docked solutions for complexes wherein CRD of SP-D bound to gp120 via glycan mediated interactions (Figure S1). The shortlisted docked poses were further refined by FireDock and the best pose was selected for further analysis (Table S1).

**Figure 2:**
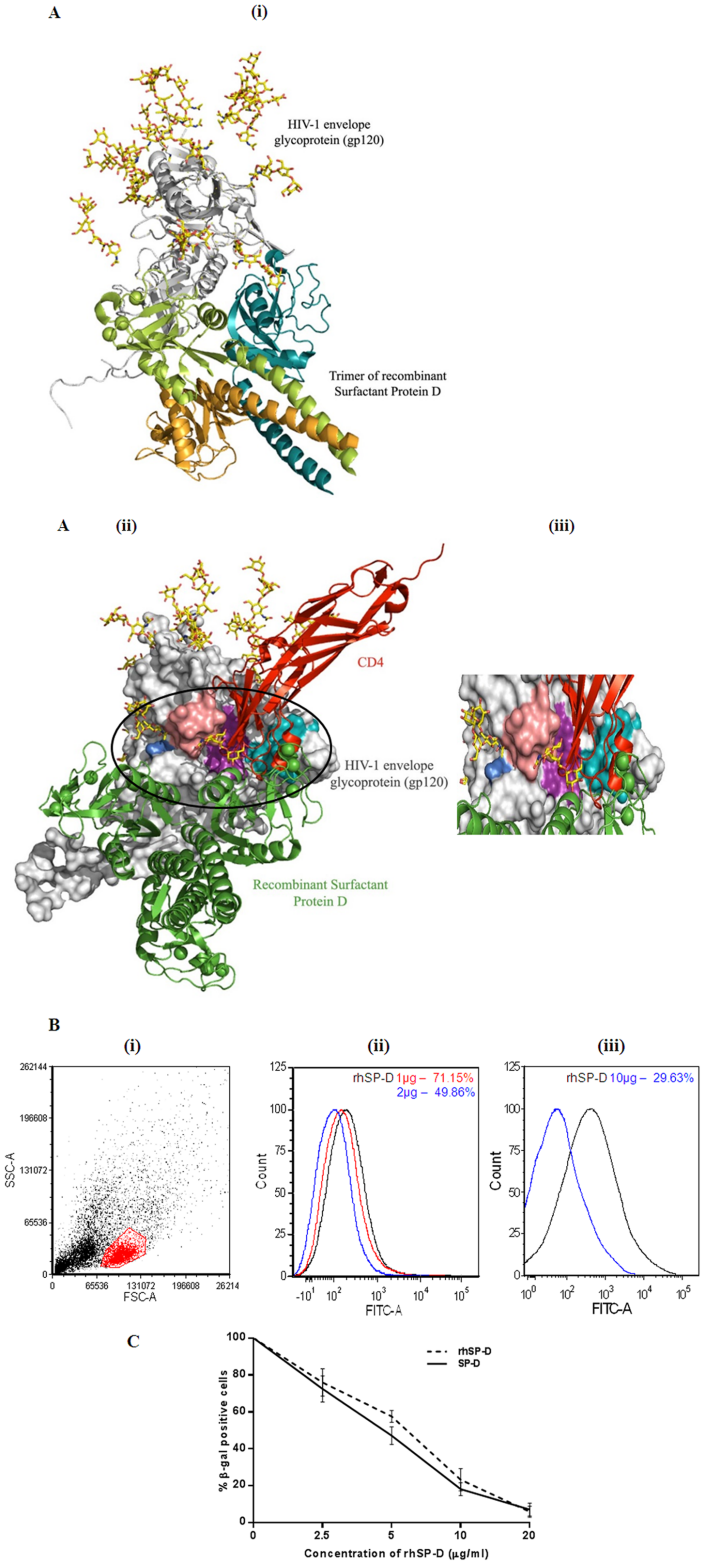
SP-D and rhSP-D interfere with binding of gp120 to CD4. (**A**) Docked solution displaying SP-D timer docked with glycosylated model of HIV-1 glycoprotein gp120. (**i**) SP-D trimer (light green, dark green and orange cartoon) interacting with HIV-1 gp120 (grey cartoon) (**ii**) SP-D trimer (green) bound proximal to the CD4 binding region of gp120 (PDB ID: 1GC1). The CD4 (red cartoon) binding regions C2 (D loop), C4 and C5 of gp120 are depicted as light red, purple and cyan surfaces, respectively. Asn234 is depicted in blue and Asn276, a part of C2 domain is depicted in light red. (**iii**) Enlarged image of site where SP-D and CD4 interact on gp120 (**B**) To assess whether SP-D or rhSP-D interfered with gp120 and CD4 binding, PBMCs were incubated together with r-gp120 (2 µg/mL) and SP-D or rhSP-D (1 and 2 µg/ml) in 5 mM CaCl_2_. The interaction was probed using a polyclonal FITC-tagged anti-gp120 antibody. Cells were acquired on a flow cytometer. (**i**) Lymphocyte population was gated. Differences in the FITC positive gated lymphocytes were calculated in terms of % inhibition. (**ii**) Mean fluorescence intensity was converted to % FITC positive cells. rhSP-D (at 1 µg 74.32±0.5%, at 2 µg 48.32±3.2%) showed a significant reduction in FITC positive cells as compared to untreated control. (iii) rhSP-D (at 10 µg–28.73±2.8%) showed a significant reduction in FITC positive cells as compared to untreated control. The data is a representative image of 4 independent experiments. A similar experiment was conducted using SP-D (data not shown) (**C**) Fusion experiment where HL2/3 cells that express gp120 were incubated with indicated concentrations rhSP-D or SP-D in presence of 5 mM CaCl_2_. These cells were fused with TZM-bl cells that express CD4. The interaction of gp120 and CD4 is directly proportional to the blue color foci observed under a light microscope in TZM-bl cells. Each data point represents the mean ± S.D. (n = 3). Presence of SP-D and rhSP-D significantly decreased gp120 and CD4 binding and further β-gal positive TZM-bl cells.

An analysis of the best docked complex revealed that SP-D (CRD) interacts with glycans at Asn234 and Asn276 and various residues in the known CD4 binding regions namely C2, C4 and C5 domains of gp120 [19] (Fig. 2Ai, ii). Recently, it has been reported that 8ANC195, which is an anti-gp120 glycan-dependent antibody, requires intact N-linked glycosylation sites of gp120 at Asn234 and Asn276 for sustaining its neutralizing activity. These glycosylation sites are known to be conserved and are proximal to CD4 binding site [20]. Interestingly, it was observed that SP-D (CRD) interacts with the glycans of these residues in the docked complex. The experimental and *in silico* observations appear to suggest that SP-D could impact binding of CD4 to gp120 and could therefore serve as a potent viral entry inhibitor.

### SP-D and rhSP-D inhibit gp120-CD4 interaction and act as cell entry inhibitor of HIV-1

We further carried out a competitive assay to assess whether SP-D or rhSP-D inhibited binding of gp120 to CD4 expressing lymphocytes, using FITC labeled polyclonal antibody to gp120. Percentage gp120-bound lymphocytes (FITC positive cells) were significantly reduced in the presence of rhSP-D (at 1 µg; 74.32±0.5%, at 2 µg 48.32±3.2%), compared to untreated control (taken as 100%) (Fig 2B ii). At concentration of 10 µg, percentage of FITC positive cells reduced to 28.73±2.8%. A similar dose dependent decrease was observed with SP-D (at 1 µg/ml; 68.81±2.7%, at 2 µg/ml 32.74±1.7%).

A cell-based assay, where gp120 expressing HL2/3 cells were fused with CD4 expressing TZM-bl cells, was performed to further confirm SP-D or rhSP-D mediated inhibition of gp120-CD4 interaction. Such an assay system is helpful in determining entry inhibitors of HIV-1. SP-D as well as rhSP-D showed dose-dependent decrease in the numbers of β-gal positive cells or virus infected cells (Fig 2C). At a concentration of 20 µg/ml, SP-D or rhSP-D showed only 7.03±2.03% and 5.9±1.76% blue stained cells, respectively, when compared with control (taken as 100%).

### Anti-HIV-1 activity of SP-D

**Figure 3:**
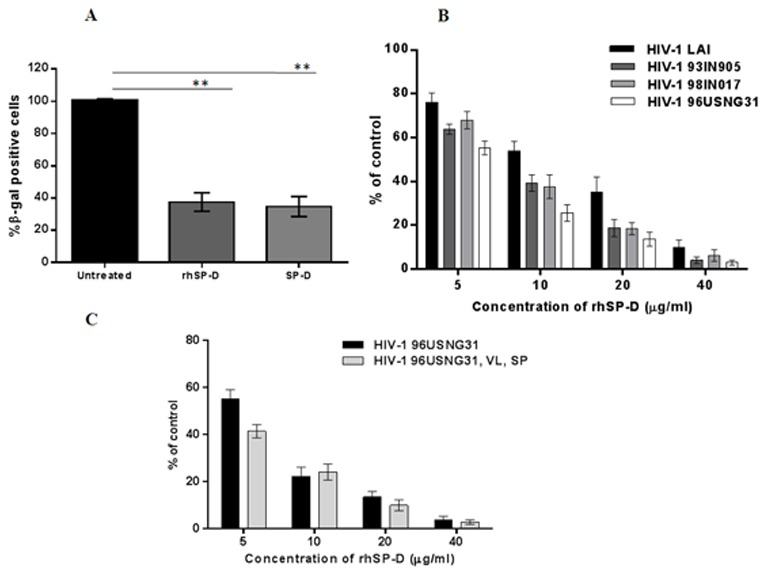
Broad spectrum anti-HIV activity of rhSP-D. (**A**) TZM-bl indicator assay to assess anti-HIV activity using rhSP-D and full-length SP-D (20 µg/ml). Each bar represents the mean ± SEM (n = 4). ** shows statistical significance in the reduction of β-gal positive cells compared to the untreated control (**p<0.01). **B**) TZM-bl indicator assay to assess anti-HIV activity of rhSP-D with different HIV-1 isolates and with various indicated concentrations. Each bar represents the mean ± SEM. (n = 4). rhSP-D showed a significant (p<0.05) reduction of β-gal positive cells as compared to the untreated control **C**) Post-coital efficacy of rhSP-D against HIV-1 using TZM-bl indicator assay. Diluted CVL was pre-incubated with indicated concentrations of rhSP-D for 1 h. 100 TCID50 of HIV-1 USNG31 were pre-incubated with undiluted SP-D for 1 h. These were mixed, incubated, and later added to TZM-bl cells. Each bar represents the mean ± SEM. (n = 4). rhSP-D showed significant (p<0.05) reduction of β-gal positive cells as compared to the untreated control.

To evaluate anti-HIV activity of SP-D, we used the TZM-bl cell line which is highly sensitive to infection with a range of HIV-1 isolates. SP-D and rhSP-D showed a significant reduction in infectivity down to 34.72±3.11% and 37.6±5.76%, respectively, as compared to control cells (taken as 100%) (Fig 3A). The reduction in infectivity observed at the same concentration of rhSP-D and SP-D was not significantly different and could be due to a considerably higher molar concentration of rhSP-D (1 µg∼16.7×10^−3^ µM) than SP-D (1 µg∼1.9×10^−3^ µM). Subsequent experiments were carried out using rhSP-D only.

### Anti-HIV-1 activity of rhSP-D is independent of viral tropism and is intact in the presence of CVL and SP

Various HIV-1 strains/isolates exhibit different level of tropism that is in part determined by glycosylation level/pattern [21]. Since SP-D is a carbohydrate pattern recognition molecule, we sought to examine the ability of rhSP-D to inhibit four different HIV-1 isolates with distinct tropism. The strains included one laboratory strain, HIV-LAI that utilizes X4, and three primary isolates: R5 tropic 93IN905, X4 tropic 98IN017, and R5X4R3 tropic 96USNG31. A significant dose-dependent rhSP-D-mediated inhibition of viral infection was observed with all the four viral strains (Fig 3B). The IC_50_ values did not vary significantly with differences in viral tropism or subtype (Table 1). Cell viability was evaluated to confirm that reduction in viral replication was not due to cell death (Fig S2A).

**Table 1:** Summary of the calculated IC50 values for rhSP-D-mediated inhibition of HIV-1 with envelopes from subtypes B and C and different cell types.

Cell type	Assay	Viral Strain	Subtype	Tropism	IC_50 (µg/ml)_
TZM-bl	Reporter Assay	HIV-1 LAI(BRU)	B	X4	10.87±0.5
TZM-bl	Reporter Assay	HIV-1 93IN905	C	R5	6.72±0.63
TZM-bl	Reporter Assay	HIV-1 98IN017	C	X4	8.95±0.51
TZM-bl	Reporter Assay	HIV-1 96USNG31	C	R5X4R3	7.26±0.52
PBMCs	p24 Antigen ELISA	HIV-1 96USNG31	C	R5X4R3	9.67±2.6
Jurkat T cells	p24 Antigen ELISA	HIV-1 96USNG31	C	R5X4R3	13.67±3.37
U937 monocytes	p24 Antigen ELISA	HIV-1 96USNG31	C	R5X4R3	10.22±1.37

To examine efficacy of rhSP-D in post-coital conditions, we studied anti-HIV activity of rhSP-D in the presence of biological fluids such as CVL and SP, which differ in terms of pH, secretory proteins and proteases. We observed that the anti-HIV-1 activity of rhSP-D remained intact in the presence of CVL and SP (Fig 3C). Since SP-D is present in vaginal mucosa and tract [16], it was expected that SP-D may retain its functions in these secretions.

### rhSP-D inhibits HIV-1 infection in Jurkat and U937 cell lines as well as activated PBMCs

**Figure 4:**
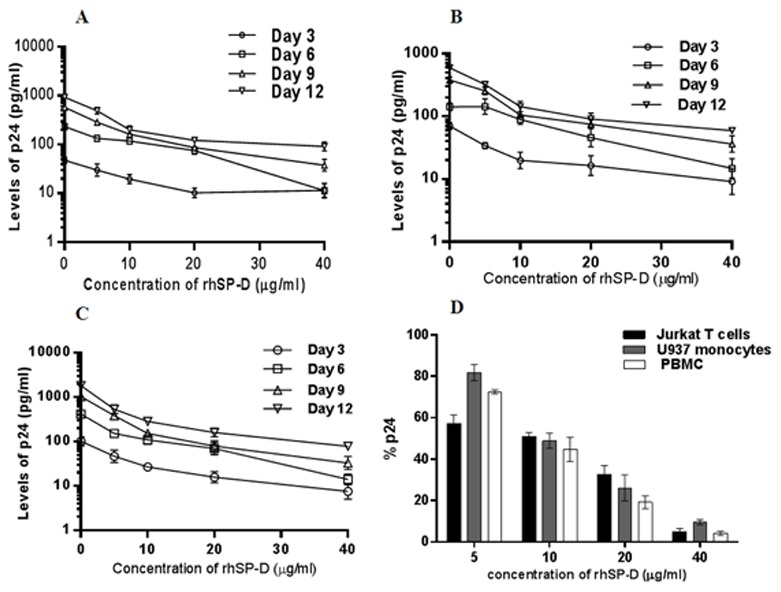
rhSP-D inhibits HIV-1 infection of Jurkat T cells, U937 monocytic cells and activated PBMCs. 100 TCID_50_ of HIV-1 USNG31 and indicated concentrations of rhSP-D in presence of 5 mM CaCl_2_ were incubated for 1 h. The virus-collectin complex was then added to cells for 4 h and unbound residual virus was removed. Fresh media was added to the cells and a sample of culture supernatant was taken every 3 days for p24 quantification by ELISA and replaced with new media. On day 12, viability was assessed by an MTT assay, which indicated no cellular death (Figure S2B). (**A**) Dose-dependent inhibition of HIV infection in Jurkat T cells; (**B**) Dose-dependent inhibition of HIV infection in U937 monocytic cells; (**C**) Dose-dependent inhibition of HIV-1 infection in PHA-activated PBMCs. Each data point represents the mean ± S.D. (n = 3). rhSP-D showed significant (p<0.05) reduction of β-gal positive cells as compared to untreated control. (**D**) Day 6 culture supernatants of Jurkat T cells, U937 monocytic cells and activated PBMCs were used to determine the concentration for a 50% reduction in p24 production in each cell type. Each bar represents the mean ± S.D. (n = 3).

CD4 T cells and monocyte/macrophages are the primary targets of HIV infection and activated PBMCs are known to be more susceptible to HIV infection. Thus, we assessed the anti-HIV activity of rhSP-D in Jurkat T cells, U937 monocytic cell line and activated PBMCs. As shown in Fig 4 (A–C), rhSP-D significantly inhibited HIV-1 infection in all three target cells tested in a dose-dependent manner over a period of 12 days. Furthermore, during the assay period, cellular viability was not affected in the presence of rhSP-D, as determined by MTT assay performed on day12 (Fig S2B), suggesting that the reduced infectivity was not due to the cellular death. We did not observe a significant difference in the inhibitory activity of rhSP-D between different cell types and the activity was persistent even with primary cells (PBMCs) as HIV-1 target.

### rhSP-D significantly inhibits pro-inflammatory cytokine production by HIV-1 challenged Jurkat T cells, U937 monocytes and activated PBMCs

**Figure 5:**
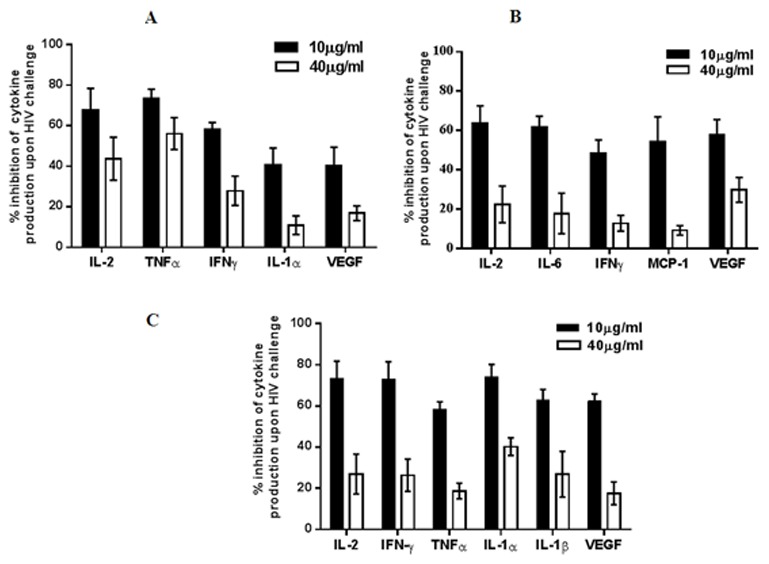
rhSP-D inhibits pro-inflammatory cytokine production of HIV-1 challenged Jurkat T cells, U937 monocytic cells and activated PBMCs. Jurkat T cells, U937 monocytes and activated PBMCs were treated with indicated concentrations of rhSP-D, 20 min prior to HIV-1 challenge. 24 h post viral challenge, culture supernatant were collected and assayed for cytokine levels by a multiplex system. (**A**) Dose-dependent % inhibition of rhSP-D-treated HIV-1 challenged cytokines IL-2, TNF-α, IFN-γ, IL-1α, VEGF as compared to HIV alone in Jurkat T cells. Each bar represents the mean ± S.D. (n = 3). rhSP-D showed significant (p<0.05) reduction in the levels of these pro-inflammatory cytokines. (**B**) Dose-dependent % inhibition of rhSP-D-treated HIV-1 challenged cytokines IL-2, IL-6, IFN-γ, MCP-1, VEGF as compared to HIV alone in U937 monocytes. Each bar represents the mean ± S.D. (n = 3). rhSP-D showed significant (p<0.05) reduction in the levels of these pro-inflammatory cytokines. (**C**) Dose-dependent % inhibition of rhSP-D treated HIV challenged cytokines IL-2, IFN-γ, TNF-α, IL-1α, IL-1β, VEGF as compared to HIV alone. Each bar represents the mean ± S.D. (n = 3). rhSP-D showed significant (p<0.05) reduction in the levels of these pro-inflammatory cytokines.

Cytokine response of the HIV challenged immune cells was determined with or without rhSP-D. There was a significant rise in the levels of pro-inflammatory cytokines such as IL-1α, IL-1β, IL-2, IL-6, IL-8, TNF-α, VEGF, IFN-γ and MCP-1 in U937 monocytes, Jurkat T cells and PBMCs after 24h of HIV-1 challenge (Table S2, S3, S4). Levels of IL-2, IFN-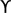 and VEGF were considerably reduced in the rhSP-D treated Jurkat, U937 and primary cells (PBMCs) in a dose-dependent manner. (Fig 5A, B, C). Levels of IL-1α and TNF-α were also significantly lowered in Jurkat cells (Fig 5A). Similarly, IL-6, a major pro-inflammatory cytokine in HIV infection, and a chemokine MCP-1, were reduced in U937 monocytes (Fig 5B). Additionally, PBMCs also showed a decrease in IL-6, MCP-1 and IL-1β (Fig 5C). Other cytokines (Table S1) that were up-regulated upon HIV-1 challenge were not significantly altered by rhSP-D. This indicates that rhSP-D successfully controls most of the pro-inflammatory cytokine production induced by HIV-1. Viability of uninfected and infected cells was not affected at 24 h (Fig S2C).

### rhSP-D modulates the phosphorylation of Akt and MAPKs in HIV-1 challenged Jurkat T cells and U937 monocytes

**Figure 6:**
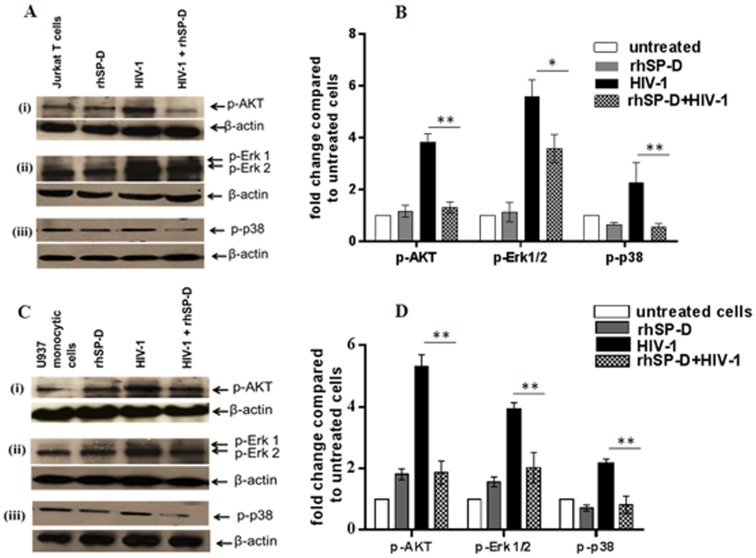
rhSP-D modulates the phosphorylation of Akt and MAPKs upon HIV-1 challenge. 5×10^5^ Jurkat T cells or U937 monocytic cells were treated with rhSP-D (10 µg/ml) in presence of 5 mM CaCl_2_ and HIV-1USNG31. After 30 min, cell were harvested, lysed, and SDS-PAGE and Western blots were performed using pAKT, p-p38 and p-Erk1/2 or β-actin (house-keeping control) antibodies. Representative Western blots (**A**) **and** (**C**) and bar graphs (**B**) **and** (**D**), generated by densitometric analysis for Jurkat and U937 cells, respectively. Each bar represents the mean ± S.D. (n = 3). *, ** shows statistical significant % inhibition cytokine levels of HIV-1 infection by rhSP-D in each cell type as compared to control (*p<0.05, **p<0.01) represent cumulative data.

PI3K activation can lead to the phosphorylation and activation of Akt [22], [23]. PI3K/Akt pathway is critical for regulating secretion of pro-inflammatory cytokines that promote HIV-1 infection [24]. Phosphorylation of Akt was significantly reduced in the rhSP-D treated T cells and monocytes. Like Akt, phosphorylation of both p38 and Erk1/2 MAPK was significantly decreased in rhSP-D treated cells, indicating the contribution of rhSP-D restricted signal transduction in bringing down the cytokine response to HIV (Fig 6).

## Discussion

Here, we show the anti-HIV-1 activity of a recombinant fragment of human SP-D containing trimeric CRDs (rhSP-D) and its ability to contain the viral replication in Jurkat T cells, U937 monocytes and activated PBMCs *in vitro*. We also demonstrate that rhSP-D, similar to full length human SP-D, recognizes the envelope glycoprotein gp120 that binds to CD4, initiating an intracellular signaling cascade that sets up viral infection in target cells. Importantly, both rhSP-D and SP-D inhibited interaction between gp120 and CD4 in a dose-responsive manner and downregulated the spiraling immune activation.

Interaction of rhSP-D with gp120 is a calcium dependent, lectin activity of the CRD similar to that of SP-D reported previously [14], [15]. It is likely that SP-D (and rhSP-D) interacts with gp41 and gp160 in a similar manner through its lectin domains. HIV-1 gp120 interacts with CD4 and co-receptors CXCR4 or CCR5 whereas gp41 assists in the fusion of the viral membrane to the cell membrane [25]. Thus, SP-D may regulate viral binding and fusion with the target cells. Consistent with this notion, both SP-D and rhSP-D inhibited the interaction between solid-phase or surface expressed gp120 and CD4. This is in contrast to a recent paper that reported that SP-D failed to inhibit the gp120-CD4 interaction in solution phase [15]. The differences in the two results ([15] and this study) may reflect on the assay systems used. It is also likely that steric hindrance imposed by rhSP-D bound gp120 may specifically inhibit interaction with membrane bound CD4. The steric context may be somewhat different for virion gp120 binding to soluble CD4 compared to membrane bound CD4, as shown for Cyanovirin-N, a potent gp120 binding lectin and an entry inhibitor [26], [27]. Inhibition of gp120-CD4 interaction implies that SP-D could affect the gp120 mediated viral entry. Analysis of the docked complex revealed that the binding regions of CRD of SP-D and CD4 in gp120 are proximal, and therefore, it is highly likely that SP-D (CRD) can impair/inhibit CD4 binding of gp120. Furthermore, the glycans of Asn234 and Asn276 are implicated in the neutralizing activity of 8ANC195 antibody, which is an anti-gp120 antibody, secreted by sorted single B cell from a patient with high titers of broadly neutralizing antibodies that bind to an HIV gp120 core glycoprotein stabilized in the CD4-bound conformation and lacking the variable (V loops 1 to 3) (2CC core) [20], [28]. Asn234 seems to be relevant for viral infectivity as exposure to an increasing concentration of *Urtica dioica* agglutinin (UDA), a carbohydrate binding agent, results in a selection pressure on Asn234 to Ile234 and the mutant virus showed reduced infectivity [29]. Glycosylated Asn276 is also a contact site for the broadly neutralizing antibody VRC01 [30]. SP-D shared interactions with the glycans of these residues in the docked complex, further strengthening its potential as a viral entry inhibitor. The gp120-CD4 fusion assay using HL2/3 cells corroborated that SP-D and rhSP-D act as potent cell entry inhibitors to HIV-1.

Binding of gp120 to CD4 on the surface of T cells, DCs, and macrophages result in the production of cytokines such as IL-6, IL-10, TNF-α, IFN-α, IFN-γ and IL-1β [31]. A recent report has shown that individuals with detectable gp120 had higher levels of plasma IL-6, IL-10, and TNF-α [32]. A number of *in vitro* and *in vivo* studies have correlated the elevated levels of pro-inflammatory cytokine/chemokine during acute HIV infection with increased viral load and enhanced pathogenesis [1], [3], [33]–[40]. We also observed a significant increase of pro-inflammatory cytokines when HIV-1 was challenged to PBMCs, U937 monocytes and Jurkat T cells *in vitro*. Since rhSP-D interferes with gp120/CD4 interaction, it can block the gp120-CD4 triggered rise of pro-inflammatory cytokines in HIV challenged cells. In the case of U937 cells, rhSP-D significantly reduced levels of IL-2, IL-6, IFN-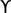, VEGF and MCP-1. Similarly rhSP-D treatment lowered expression of IL-2, TNF-α, IFN-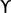, IL-1α and VEGF in HIV infected Jurkat cells. Activated PBMCs showed a significant decrease in cytokines including, IL-2, IFN-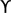, TNF-α, IL-1α, IL-1β and VEGF. Since these cytokines are involved in non-specific immune activation, infiltration of cells and viral replication, SP-D may be playing a vital role in modulating the acute phase immune response to HIV challenge. Such a controlled cytokine milieu may aid in reducing the susceptibility of target cells to infection and facilitate a protective anti-HIV response. Similarly, SP-D has been shown to be protective and anti-inflammatory in mouse models of other viral infections like Influenza A Virus and Respiratory Syncytial Virus [41], [42].

Binding of HIV-1 to CD4 activates the MEK/Erk kinase pathway, stimulates the expression of nuclear factors, and results in the expression of inflammatory genes [43]. Signaling through p38 and Erk pathways could be a common link between HIV replication and immune response [44]. HIV infection also activates cellular PI3/Akt pathway that promote viral replication. The PI3-kinase effectors Akt and p70 S6 kinase have been shown to phosphorylate in response to both soluble and virion-associated R5 and X4 gp120 in primary CD4 T lymphocytes [24]. A rhSP-D mediated interference between gp120 and CD4 interaction probably explains a significant reduction in phosphorylation of Akt, p38 and Erk1/2 in HIV-1 challenged Jurkat and U937 cells. Thus, in addition to inhibiting the gp120 mediated virion entry into the target cells, SP-D also dampens activation of signaling pathways, leading to modulation of pro-inflammatory cytokine production by target cells. Having used four HIV strains that differ with respect to subtypes (env) B and C and X4, R5 or dual tropic strains, we infer that the inhibitory activity of rhSP-D against HIV-1 is broad, independent of tropism, dose-dependent, and effective against subtype B and C envelope sequences. This could be, in part, attributed to presence of well conserved mannosylated domains of gp120 among various virus subtypes, an advantage associated with agents targeting HIV glycans [45].

Considering that more than 85% of HIV transmission occurs due to heterosexual contact and SP-D is present in the vaginal tract [16], we sought to assess the post-coital efficacy of rhSP-D to inhibit HIV infectivity. An effective inhibition of HIV-1 infection in TZM-bl cells was observed in presence of CVL and SP. SP-D has been reported to be present in the vaginal tract and is localized on human spermatozoa and seminal plasma of stallion [46], [47]. Thus, it was unlikely to be inhibited or inactivated in these fluids. Our results lend support to the idea that SP-D plays an important role in curbing the post-coital HIV-1 infection and transmission *in vivo*. This raises the possibility that rhSP-D can be effectively used as a prophylactic intervention strategy at the mucosal level.

In conclusion, the study has enabled us to assign specific roles to SP-D in host defense against HIV. SP-D appears to play a protective role by enhancing viral clearance via its recognition of the glycosylated envelope of HIV-1, inhibiting viral entry by blocking access of envelope glycoproteins to the cell surface receptors, and thus, interfering with virus internalization by host cells as well as with reduced signal transduction, immune cell activation and pro-inflammatory cytokine production, resulting in reduced viral loads. As genital mucosal compartment is the usual portal of entry for HIV and other STI pathogens, the local production of SP-D is likely to play a role in innate defense responses against the virus. The study emphasizes the potential of rhSP-D for translational applications in the HIV related healthcare.

## Materials and Methods

### Ethics Statement

Written informed consent was obtained from each study participant and recommended guidelines were followed during sample collection. The study was approved by the Institutional Ethics Committee for Clinical Studies, National Institute for Research in Reproductive Health (ICMR); (Project 148/2008), and recommended guidelines were followed during collection of blood, vaginal lavage and seminal plasma from the study participants. For purification of SP-D from the amniotic fluid collected at term, the study was approved by the Institutional Ethics Committee for Clinical Studies, National Institute for Research in Reproductive Health (ICMR); (Project 153/2009), and Ethics Committee of KEM Hospital and Seth GS Medical College; Project (EC/GOVT-6/2009).

### Viral strains

Four HIV-1 strains namely LAI (X4 tropic), 93IN905 (R5 tropic, env. subtype C), 98IN017 (utilizes X4, env. subtype C), 96USNG31 (utilizes R5X4R3, env. subtype C) were obtained from NIH-AIDS Research and Reference Reagent Program, USA.

### PBMCs and Cell lines

TZM-bl and HL2/3 cell lines were obtained from NIH-AIDS Research and Reference Reagent Program, USA whereas, Jurkat T and U937 monocytic cell lines were procured from Cell Repository Facility, National Center for Cell Sciences, Pune, India. TZM-bl cells were cultured in DMEM/F12 medium (Sigma) containing 10% FBS (Gibco) and 1% Pen-Strep (Gibco). HL2/3 cells were cultured in complete DMEM/F12 containing G418 (Geneticin from Gibco) at a concentration of 50 µg/ml. Jurkat T and U937 monocytic cell lines were cultured in RPMI-1640 medium (Sigma). Cells were sub-cultured every 2-3 days and those at the log phase of growth were used for the experiments.

Peripheral Blood Mononuclear Cells (PBMCs) pooled from different study participants were procured from Himedia Laboratories. Cells were cultured in RPMI 1640 medium containing 10% FBS (Gibco) in the presence of phytohemaglutinin (PHA) (Gibco). PHA was washed off after 24 h and cells were cultured further in complete RPMI medium.

### Collection of Cervico-vaginal lavage (CVL)

After obtaining written informed consent, proliferative phase CVL from normal cycling females (25–32 years; n = 5) was collected by placing a #2 Pederson speculum in the vagina and instilling 3 ml 0.9% sterile saline into the speculum. A 5 ml syringe was used to rinse vaginal walls once, and the lavage fluid was then withdrawn from the speculum. Lavage was passed through a syringe filter (0.22 micron) and was frozen at −80°C immediately.

### Collection of Seminal plasma (SP)

Written consent was obtained from healthy males, age (27–35; n = 5) and semen sample was collected. Sperms were pelleted and seminal plasma was recovered and stored at −80°C.

### hSP-D preparation

Native human SP-D (hSP-D) was purified from the human amniotic fluid obtained from pregnant women at term undergoing caesarean section, using procedures described previously [48], [49]. The protein preparation was confirmed to be pure by SDS-PAGE and western blot analysis.

### Recombinant form of SP-D preparation

The rhSP-D was expressed in *E. coli*, purified and characterized as described previously [50]. The rhSP-D preparation was examined for endotoxin levels using the QCL-1000 Limulus amebocyte lysate system (BioWhittaker Inc., USA). The assay was linear over a range of 0.1–1.0 EU/ml (10 EU = 1 ng of endotoxin) and the amount of endotoxin present in the preparations was estimated to be 4 pg/µg of rhSP-D.

### Study on interactions of SP-D and gp120

#### (A) Ligand Blot analysis

HIV antigen PVDF strips (MP Diagnostics) were probed with 1 µg/ml of SP-D or rhSP-D in Tris buffer saline (TBS, 0.01 M, pH 7.4) containing 5 mM CaCl_2_ at room temperature for 1 h. The strip was washed three times with TBS containing 0.05% Tween-20 followed by incubation with 1∶100 diluted monoclonal antibody against human SP-D (Abcam). After washing, the strip was incubated with 1∶1000 anti-mouse IgG-peroxidase (Dako) and was developed using ECL detection system.

#### (B) ELISA to examine gp120-SP-D interaction

2 µg r-gp120 (Xpress Bio) was immobilized on 96-well Maxisorp plates (Nunc) in 0.1 M sodium bicarbonate buffer (pH 9.6) overnight at 4°C. The wells were washed with PBS and 0.05% (v/v) Tween 20 (PBST) and blocked in 3% (v/v) BSA for 1 h at 37°C. After washing away excess BSA with PBST, the wells were incubated with increasing concentrations of SP-D or rhSP-D (0–10 µg/ml) in Tris buffer saline calcium (TBSC; 20 mM Tris-HCl, 150 mM NaCl, 5 mM CaCl_2_, pH 7.4), or Tris-saline EDTA (TBSE; 20 mM Tris-HCl, 150 mM NaCl, 2 mM EDTA, pH 7.4). The gp120-SP-D/rhSP-D complexes were detected using a biotinylated polyclonal antibody for SP-D (Abcam) and HRP-Streptavidin (Santa Cruz Antibodies Inc.) and H_2_O_2_-TMB (Sigma). The absorbance (450 nm) of individual wells was measured by a spectrophotometer (Beckman Coulter).

#### (C) Binding of SP-D to cell surface expressed gp120 on HL2/3 cells

HeLa-derived HL2/3 cells, which express HIV-1HXB2 Env on the cell surface in addition to Tat, Gag, Rev, and Nef proteins in the cytoplasm [51], were treated with or without SP-D or rhSP-D (2 µg/ml) on ice for 1 h. Cells were then labeled with anti-gp120/FITC antibody. Differences in FITC positive cells in control versus treated were analyzed by FACS (BD FACS Aria III).

#### (D) Protein Docking

The structures of human lung surfactant protein D (PDB ID: 1PW9) [52] in the trimeric form and HIV-1 envelope glycoprotein gp120 complexed with two-domain fragment of human CD4 receptor (PDB ID: 1GC1) modeled with glycans [53] were docked using PatchDock server with default parameters [54]. The shortlisted poses were refined using FireDock [55].

### Effect of SP-D on interaction between gp120 with CD4

#### (A) Competitive inhibition assay for soluble gp120 binding to CD4 on lymphocytes

To assess whether SP-D or rhSP-D intervention inhibited the interaction between gp120 and CD4, PBMCs were incubated with or without SP-D or rhSP-D (1, 2 µg/ml) and gp120 (2 µg) on ice for 45 min. Cells were then probed with anti-gp120/FITC antibody (Fitzgerald, USA). Lymphocyte population was gated and FITC positive cells were analyzed immediately by FACS.

#### (B) Assay for cell surface expressed gp120 dependent entry inhibition

HL2/3 cells were co-cultured with TZM-bl cells that express CD4 and CXCR4/CCR5, respectively, at a 1∶1 cell density ratio (2×10^4^ cell/well each) for 18 h with or without SP-D or rhSP-D (0–20 µg/ml) in order to examine whether SP-D or rhSP-D interferes with the binding of HIV-1 *Env* with cell surface receptor/co-receptor. Upon fusion of TZM-bl and HL2/3 cells, Tat protein from HL2/3 cells activates β-galactosidase indicator gene expression in TZM-bl cells. The fused cells, seen as blue foci, were counted under a light microscope. The number of blue colored foci is directly proportional to the fused cells, and thus, gp120-CD4 interaction can be assessed.

### Anti-HIV assays

#### (A) Indicator Assay

HIV-1 infection was quantified using TZM-bl cells, which express luciferase as well as β-galactosidase genes under the control of HIV-1 LTR promoter [56]. TZM-bl cells (6×10^3^) were grown in a 96-well tissue culture plate for 24 h. In separate tubes, 100 TCID50 units of HIV-1 (strains: HIV-1LAI, HIV-1 IN93/905, HIV-1 98/IN/017 and HIV-1 96USNG31) were pretreated with indicated concentrations of rhSP-D in presence 5 mM CaCl_2_ for 1 h at 37°C. The rhSP-D opsonized virus was allowed to infect TZM-bl cells. After 4 h, excess virus was washed with 50 mM PBS, fresh medium was added, and then cells were incubated for an additional 48 h. Next, cells were washed twice with PBS, fixed in 1% glutaraldehyde for 10 min at room temperature, followed by treatment with X-gal staining solution (10 ml PBS with 1 mg/ml X-gal dissolved in DMF, 100 mM potassium ferricyanide, 100 mM potassium ferrocyanide and 1 mM MgCl_2_) for 24 h at 37°C. The blue stained (β-gal expressing) foci were counted under the microscope. In a parallel experiment, viability of cells was determined by MTT assay after 48 h as described earlier [57].

#### (B) Anti-HIV activity in presence of CVL and SP

Majority of the HIV transmission occurs due to heterosexual contact. Thus, it was important to re-confirm the anti-HIV activity of rhSP-D in presence of vaginal lavage and seminal plasma. To evaluate the post-coital efficacy of anti-HIV activity of rhSP-D, 100 TCID_50_ of HIV-1 96USNG31 (R5X4R3 tropic) was mixed with 50 µl of undiluted seminal plasma and incubated for 1 h. The rhSP-D at indicated concentrations in presence of 5 mM CaCl_2_ was mixed with 100 µl diluted CVL (pH 5) and incubated for 1 h. Later, the virus in the seminal plasma and the rhSP-D in the lavage were mixed and incubated for 2 h [58]. The experimental conditions were created to mimic the vaginal milieu during sexual contact. Further, the pretreated virus with CVL and SP was added to TZM-bl cells and the assay was carried out as described above.

#### (C) Quantitation of p24 levels in culture supernatants

A day prior to viral challenge, 5×10^4^ of Jurkat, U937, or PHA activated PBMCs were incubated in RPMI 1640 medium containing 10% FBS. Different concentrations of rhSP-D in presence of 5 mM CaCl_2_ were pre-incubated with 100 TCID_50_ of HIV-1 96USNG31 for 1 h before being exposed to target cells. After 4 h, residual virus was removed, cells were washed and fresh medium was added. On day 3, 6, 9 and 12, 100 µl supernatant was collected and replaced with fresh media. The samples were stored at −80°C until viral replication was determined by the HIV-1 p24 Antigen Capture Assay ELISA (Xpress Bio). On day 12, MTT assay was performed to assess cellular viability. The p24 values of day 6 supernatant were used to calculate the IC_50_ concentration of rhSP-D.

### Kinase activity and cytokine analysis

U937 or Jurkat cells (5×10^5^ cells) were seeded in a 24-well plate overnight. Cells were then treated with rhSP-D in presence of 5 mM CaCl_2_ at the concentration of 10 and 40 µg/ml for 20 min prior to HIV-1 challenge. After HIV challenge, cells were harvested after 30 min for analyzing phosphorylation of Erk1/2, p38 and AKT (Cell Signaling). Subsequently, 24 h culture supernatants were collected for cytokine analysis using Cytokine Array 1 kit for IL-1α, IL-1β, IL-2, IL-4, IL-6, IL-8, IL-10, IFN-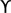, TNF-α, MCP-1, VEGF, EGF on Evidence Investigator, a multiplex system that uses Biochip Array Technology (Randox Laboratories).

### Statistical Analysis

Data are expressed as Mean ± SEM. Dose response relationships were evaluated using one-way ANOVA, and post-hoc comparisons between individual treatments were made using Tukey's test. Instances involving only two comparisons were evaluated by paired Student's t-tests. A value of p<0.05 was considered statistically significant.

## Supporting Information

Figure S1 **Selected Docked Poses.** Patchdock poses (**i**), (**ii**) and (**iii**) exhibiting the CRD of SP-D trimer (light green, dark green and orange cartoon) interacting via glycans modeled on glycoprotein gp120 (grey cartoon) were further refined by FireDock. Best Pose (**ii**) was further analyzed in the study.
(TIF)

Figure S2 **MTT assay to evaluate viability of rhSP-D treated uninfected and infected cells.** (**A**) Viability of TZM-bl cells treated with rhSP-D at 48 h post-infection. Each bar represents the mean ± S.D. (n = 4). (**B**) Viability of Jurkat T cells, U937 monocytes and activated PBMCs treated with rhSP-D on day 12 of infection. Each bar represents the mean ± S.D. (n = 3). (**C**) Viability of Jurkat T cells, U937 monocytes and activated PBMCs treated with rhSP-D at 24 h post HIV-1 infection. Each bar represents the mean ± S.D. (n = 3). These different time points with different cells are in coherence with the anti-HIV and differential cytokine expression assays carried out in the present study. Data suggest that viability of cells was not affected by rhSP-D during experiments for anti-HIV activity and differential cytokine expression.
(TIF)

Table S1 **Ranking and energy for solutions refined using FireDock.**
(DOCX)

Table S2 **Levels of cytokines (pg/ml) in culture supernatants of Jurkat T cells on treatment with indicated concentration of rhSP-D, HIV-1 and HIV-1 and rhSP-D.**
(DOCX)

Table S3 **Levels of cytokines (pg/ml) in culture supernatants of U937 cells on treatment with indicated concentration of rhSP-D, HIV-1 and HIV-1 and rhSP-D.**
(DOCX)

Table S4 **Levels of cytokines (pg/ml) in culture supernatants of PBMCs on treatment with indicated concentration of rhSP-D, HIV-1 and HIV-1 and rhSP-D.**
(DOCX)

## References

[1] Stacey AR, Norris PJ, Qin L, Haygreen EA, Taylor E, et al (2009) Induction of a striking systemic cytokine cascade prior to peak viremia in acute human immunodeficiency virus type 1 infection, in contrast to more modest and delayed responses in acute hepatitis B and C virus infections. J Virol 83(8): 3719–3733 10.1128/JVI.01844-08 19176632 PMC2663284

[2] Herbein G, Gras G, Khan KA, Abbas W (2010) Macrophage signaling in HIV-1 infection. Retrovirology 7: 34 10.1186/1742-4690-7-34 20380698 PMC2865443

[3] Chun TW, Engel D, Mizell SB, Ehler LA, Fauci AS (1998) Induction of HIV-1 replication in latently infected CD4+ T cells using a combination of cytokines. J Exp Med 6; 188(1): 83–91. 9653086 10.1084/jem.188.1.83 PMC2525548

[4] McMichael AJ, Borrow P, Tomaras GD, Goonetilleke N, Haynes BF (2010) The immune response during acute HIV-1 infection: clues for vaccine development. Nat. Rev. Immunol 10(1): 11–23 10.1038/nri2674 20010788 PMC3119211

[5] Nayak A, Dodagatta-Marri E, Tsolaki AG, Kishore U (2012) An Insight into the Diverse Roles of Surfactant Proteins, SP-A and SP-D in Innate and Adaptive Immunity. Front Immunol 7; 3: 131 10.3389/fimmu.2012.00131 22701116 PMC3369187

[6] Kishore U, Greenhough TJ, Waters P, Shrive AK, Ghai R, et al (2006) Surfactant proteins SP-A and SP-D: structure, function and receptors. Mol. Immunol 43(9): 1293–1315. 16213021 10.1016/j.molimm.2005.08.004

[7] Gardai SJ, Xiao YQ, Dickinson M, Nick JA, Voelker DR, et al (2003) By binding SIRPalpha or calreticulin/CD91, lung collectins act as dual function surveillance molecules to suppress or enhance inflammation. Cell 115(1): 13–23. 14531999 10.1016/s0092-8674(03)00758-x

[8] Jakel A, Qaseem AS, Kishore U, Sim RB (2013) Ligands and receptors of lung surfactant proteins SP-A and SP-D. Front Biosci 18: 1129–1140. 10.2741/4168 23747872

[9] Yamazoe M, Nishitani C, Takahashi M, Katoh T, Ariki S, et al (2008) Pulmonary surfactant protein D inhibits lipopolysaccharide (LPS)-induced inflammatory cell responses by altering LPS binding to its receptors. J Biol Chem 283(51): 35878–35888 10.1074/jbc.M807268200 18990700

[10] Borron PJ, Crouch EC, Lewis JF, Wright JR, Possmayer F, et al (1998) Recombinant rat surfactant-associated protein D inhibits human T lymphocyte proliferation and IL-2 production. J Immunol 161(9): 4599–4603. 9794387

[11] Borron PJ, Mostaghel EA, Doyle C, Walsh ES, McHeyzer-Williams MG, et al (2002) Pulmonary surfactant proteins A and D directly suppress CD3+/CD4+ cell function: evidence for two shared mechanisms. J Immunol 169(10): 5844–5850. 12421966 10.4049/jimmunol.169.10.5844

[12] Lin KW, Jen KY, Suarez CJ, Crouch EC, Perkins DL, et al (2010) Surfactant protein D-mediated decrease of allergen-induced inflammation is dependent upon CTLA4. J Immunol 184(11): 6343–6349 10.4049/jimmunol.0901947 20435925 PMC2905687

[13] Clark H, Reid KB (2003) The potential of recombinant surfactant protein D therapy to reduce inflammation in neonatal chronic lung disease, cystic fibrosis, and emphysema. Arch Dis Child 88(11): 981–984 10.1136/adc.88.11.981 14612363 PMC1719357

[14] Meschi J, Crouch EC, Skolnik P, Yahya K, Holmskov U, et al (2005) Surfactant protein D binds to human immunodeficiency virus (HIV) envelope protein gp120 and inhibits HIV replication. J Gen Virol 86: 3097–3107. 16227233 10.1099/vir.0.80764-0

[15] Madsen J, Gaiha GD, Palaniyar N, Dong T, Mitchell DA, et al (2013) Surfactant Protein D modulates HIV infection of both T-cells and dendritic cells. PLoS One 8(3): e59047 10.1371/journal.pone.0059047 23527085 PMC3601116

[16] Leth-Larsen R, Floridon C, Nielsen O, Holmskov U (2004) Surfactant protein D in the female genital tract. Mol Hum Reprod 10: 149–154 10.1093/molehr/gah022 14981140

[17] Jambo KC, French N, Zijlstra E, Gordon SB (2009) AIDS patients have increased surfactant protein D but normal mannose binding lectin levels in lung fluid. Respir Res 13 8: 42. 10.1186/1465-9921-8-42 PMC1906751 17567900

[18] Kunisaki KM, Quick H, Baker JV (2011) HIV antiretroviral therapy reduces circulating surfactant protein-D levels. HIV Med 12(9): 580–581 10.1111/j.1468-1293.2011.00920.x 21951596 PMC3629819

[19] Li Y, O'Dell S, Walker LM, Wu X, Guenaga J, et al (2011) Mechanism of neutralization by the broadly neutralizing HIV-1 monoclonal antibody VRC01. J. Virol 85(17): 8954–8967 10.1128/JVI.00754-11 21715490 PMC3165784

[20] West AP Jr, Scharf L, Horwitz J, Klein F, Nussenzweig MC, et al (2013) Computational analysis of anti-HIV-1 antibody neutralization panel data to identify potential functional epitope residues. Proc Natl Acad Sci U S A 110(26): 10598–10603 10.1073/pnas.1309215110 23754383 PMC3696754

[21] Clevestig P, Pramanik L, Leitner T, Ehrnst A (2006) CCR5 use by human immunodeficiency virus type 1 is associated closely with the gp120 V3 loop N-linked glycosylation site. J Gen Virol 87(Pt 3): 607–612. 16476981 10.1099/vir.0.81510-0

[22] Stokoe D, Stephens LR, Copeland T, Gaffney PR, Reese CB, et al (1997) Dual role of phosphatidylinositol-3,4,5-trisphosphate in the activation of protein kinase B. Science 277: 567–570. 9228007 10.1126/science.277.5325.567

[23] Bellacosa A, Chan TO, Ahmed NN, Datta K, Malstrom S, et al (1998) Akt activation by growth factors is a multiple-step process: the role of the PH domain. Oncogene 17: 313–325. 9690513 10.1038/sj.onc.1201947

[24] François F, Klotman ME (2003) Phosphatidylinositol 3-kinase regulates human immunodeficiency virus type 1 replication following viral entry in primary CD4+ T lymphocytes and macrophages. J Virol 77(4): 2539–2549. 12551992 10.1128/JVI.77.4.2539-2549.2003 PMC141101

[25] Gallo SA, Finnegan CM, Viard M, Raviv Y, Dimitrov A, et al (2003) The HIV Env-mediated fusion reaction. Biochim Biophys Acta 1614(1): 36–50. 12873764 10.1016/s0005-2736(03)00161-5

[26] Parren PW, Mondor I, Naniche D, Ditzel HJ Klasse PJ, et al (1998) Neutralization of human immunodeficiency virus type 1 by antibody to gp120 is determined primarily by occupancy of sites on the virion irrespective of epitope specificity. J. Virol 72: 3512–3519. 9557629 10.1128/jvi.72.5.3512-3519.1998 PMC109569

[27] Esser MT, Mori T, Mondor I, Sattentau QJ, Dey B, et al (1999) Cyanovirin-N binds to gp120 to interfere with CD4-dependent human immunodeficiency virus type 1 virion binding, fusion, and infectivity but does not affect the CD4 binding site on gp120 or soluble CD4-induced conformational changes in gp120. J Virol 73(5): 4360–4371. 10196334 10.1128/jvi.73.5.4360-4371.1999 PMC104217

[28] Scheid JF, Mouquet H, Ueberheide B, Diskin R, Klein F, et al (2011) Sequence and structural convergence of broad and potent HIV antibodies that mimic CD4 binding. Science 333(6049): 1633–1637. 21764753 10.1126/science.1207227 PMC3351836

[29] Balzarini J, Van Laethem K, Hatse S, Froeyen M, Peumans W, et al (2005) Carbohydrate-binding agents cause deletions of highly conserved glycosylation sites in HIV GP120: a new therapeutic concept to hit the achilles heel of HIV. J Biol Chem 280(49): 41005–41014. 16183648 10.1074/jbc.M508801200

[30] Li Y, O'Dell S, Walker LM, Wu X, Guenaga J, et al (2011) Mechanism of neutralization by the broadly neutralizing HIV-1 monoclonal antibody VRC01. J Virol. Sep 85(17): 8954–67 10.1128/JVI.00754-11 PMC3165784 21715490

[31] Chirmule N, Pahwa S (1996) Envelope glycoproteins of human immunodeficiency virus type 1: Profound influences on immune functions. Microbiol Rev 60(2): 386–406. 8801439 10.1128/mr.60.2.386-406.1996 PMC239449

[32] Rychert J, Strick D, Bazner S, Robinson J, Rosenberg E (2010) Detection of HIV gp120 in plasma during early HIV infection is associated with increased pro-inflammatory and immunoregulatory cytokines. AIDS Res Hum Retroviruses 26(10): 1139–1145 10.1089/aid.2009.0290 20722464 PMC2982714

[33] Longo N, Zabay JM, Sempere JM, Navarro J, Fernández-Cruz E (1993) Altered production of PGE2, IL-1 beta and TNF-alpha by peripheral blood monocytes from HIV-positive individuals at early stages of HIV infection. J Acquir Immune Defic Syndr 6(9): 1017–1023. 8340891

[34] Biswas P, Poli G, Orenstein JM, Fauci AS (1994) Cytokine-mediated induction of human immunodeficiency virus (HIV) expression and cell death in chronically infected U1 cells: do tumor necrosis factor alpha and gamma interferon selectively kill HIV-infected cells? J Virol 68(4): 2598–604. 7511175 10.1128/jvi.68.4.2598-2604.1994 PMC236737

[35] Zaitseva M, Lee S, Lapham C, Taffs R, King L, et al (2000) Interferon gamma and interleukin 6 modulate the susceptibility of macrophages to human immunodeficiency virus type 1 infection. Blood 96 (9): 3109–3117. 11049991

[36] Poli G, Kinter AL, Fauci AS (1994) Interleukin 1 induces expression of the human immunodeficiency virus alone and in synergy with interleukin 6 in chronically infected U1 cells: inhibition of inductive effects by the interleukin 1 receptor antagonist. Proc Natl Acad Sci U S A 91(1): 108–112. 7506410 10.1073/pnas.91.1.108 PMC42895

[37] Graziosi C, Gantt KR, Vaccarezza M, Demarest JF, Daucher M, et al (1996) Kinetics of cytokine expression during primary human immunodeficiency virus type 1 infection. Proc Natl Acad Sci U S A 93(9): 4386–4391. 8633076 10.1073/pnas.93.9.4386 PMC39547

[38] Mengozzi M, De Filippi C, Transidico P, Biswas P, Cota M, et al (1999) Human immunodeficiency virus replication induces monocyte chemotactic protein-1 in human macrophages and U937 promonocytic cells. Blood 93(6): 1851–1857. 10068657

[39] Bahbouhi B, Landay A, Al-Harthi L (2004) Dynamics of cytokine expression in HIV productively infected primary CD4+ T cells. Blood 103(12): 4581–4587. 14764521 10.1182/blood-2003-12-4172

[40] Ascherl G, Hohenadl C, Schatz O, Shumay E, Bogner J, et al (1999) Infection with human immunodeficiency virus-1 increases expression of vascular endothelial cell growth factor in T cells: implications for acquired immunodeficiency syndrome-associated vasculopathy. Blood 93(12): 4232–4241. 10361120

[41] LeVine AM, Whitsett JA, Hartshorn KL, Crouch EC, Korfhagen TR (2001) Surfactant Protein D Enhances Clearance of Influenza A Virus from the Lung In Vivo. J Immunol 167: 5868–5873. 11698462 10.4049/jimmunol.167.10.5868

[42] LeVine AM, Elliott J, Whitsett JA, Srikiatkhachorn A, Crouch E, et al (2004) Surfactant protein-D enhances phagocytosis and pulmonary clearance of respiratory syncytial virus. Am J Respir Cell Mol Biol 31(2): 193–199. 15016617 10.1165/rcmb.2003-0107OC

[43] Popik W, Hesselger JE, Pitha PM (1998) Binding of human immunodeficiency virus type 1 to CD4 and CXCR4 receptors differentially regulates the expression of inflammatory genes and activates the MEK/ERK signaling pathway. J. Virol 72: 6404–6413. 10.1128/jvi.72.8.6406-6413.1998 PMC109793 9658081

[44] Furler RL, Uittenbogaart CH (2010) Signaling through the P38 and ERK pathways: a common link between HIV replication and the immune response. Immunol Res 2010 48(1–3): 99–109 10.1007/s12026-010-8170-1 20725863

[45] Balzarini J (2007) Carbohydrate-binding agents: a potential future cornerstone for the chemotherapy of enveloped viruses? Antivir Chem Chemother 18(1): 1–11. 17354647 10.1177/095632020701800101

[46] Kankavi O, Ata A, Celik-Ozenci C, Sati L, Ciftcioglu MA, et al (2008) Presence and subcellular localizations of surfactant proteins A and D in human spermatozoa. Fertil Steril 90(5): 1904–1909 10.1016/j.fertnstert.2007.09.064 18191856

[47] Kankavi O, Ata A, Akif Ciftcioglu M (2006) Surfactant protein A and D in the reproductive tract of stallion. Theriogenology 66(5): 1057–1064. 16620929 10.1016/j.theriogenology.2006.02.047

[48] Strong P, Kishore U, Morgan C, Lopez Bernal A, Singh M, et al (1998) A novel method of purifying lung surfactant proteins A and D from the lung lavage of alveolar proteinosis patients and from pooled amniotic fluid. J Immunol Methods 220(1–2): 139–149. 9839935 10.1016/s0022-1759(98)00160-4

[49] Dodagatta-Marri E, Qaseem AS, Karbani N, Tsolaki AG, Waters P, et al (2014) Purification of surfactant protein D (SP-D) from pooled amniotic fluid and bronchoalveolar lavage. Methods Mol Biol 1100: 273–290 10.1007/978-1-62703-724-222 24218267

[50] Mahajan L, Madan T, Kamal N, Singh VK, Sim RB, et al (2008) Recombinant surfactant protein-D selectively increases apoptosis in eosinophils of allergic asthmatics and enhances uptake of apoptotic eosinophils by macrophages. Int Immunol 2008 20(8): 993–1007 10.1093/intimm/dxn058 18628238

[51] Ciminale V, Felber BK, Campbell M, Pavlakis GN (1990) A bioassay for HIV-1 based on Env-CD4 interaction. AIDS Res. Hum.Retrovir 6: 1281–1287. 2078409 10.1089/aid.1990.6.1281

[52] Shrive AK, Tharia HA, Strong P, Kishore U, Burns I, et al (2003) High-resolution structural insights into ligand binding and immune cell recognition by human lung surfactant protein D. J Mol Biol 331(2): 509–523. 12888356 10.1016/s0022-2836(03)00761-7

[53] Pancera M, Majeed S, Ban YE, Chen L, Huang CC, et al (2010) Structure of HIV-1 gp120 with gp41-interactive region reveals layered envelope architecture and basis of conformational mobility. Proc Natl Acad Sci U S A 107(3): 1166–1171 10.1073/pnas.0911004107 20080564 PMC2824281

[54] Schneidman-Duhovny D, Inbar Y, Nussinov R, Wolfson HJ (2005) PatchDock and SymmDock: servers for rigid and symmetric docking. Nucl. Acids. Res 33: W363–367. 15980490 10.1093/nar/gki481 PMC1160241

[55] Andrusier N, Nussinov R, Wolfson HJ (2007) FireDock: Fast Interaction Refinement in Molecular Docking. Proteins 69(1): 139–159. 17598144 10.1002/prot.21495

[56] Swanson MD, Winter HC, Goldstein IJ, Markovitz DM (2010) A lectin isolated from bananas is a potent inhibitor of HIV replication. J Biol Chem 285(12): 8646–8655 10.1074/jbc.M109.034926 20080975 PMC2838287

[57] Mahajan L, Pandit H, Madan T, Gautam P, Yadav AK, et al (2013) Human surfactant protein D alters oxidative stress and HMGA1 expression to induce p53 apoptotic pathway in eosinophil leukemic cell line. PLoS One 8(12): e85046 10.1371/journal.pone.0085046 24391984 PMC3877357

[58] Keller MJ, Mesquita PM, Torres NM, Cho S, Shust G, et al (2010) Postcoital bioavailability and antiviral activity of 0.5% PRO 2000 gel: implications for future microbicide clinical trials. PLoS One 5(1): e8781 10.1371/journal.pone.0008781 20107502 PMC2809740

