# Supplementary figures and images for: Surfactant Protein D Inhibits HIV-1 Infection of Target Cells via Interference with gp120-CD4 Interaction and Modulates Pro-Inflammatory Cytokine Production
Source: PLoS One. 2014 Jul 18;9(7):e102395. doi: 10.1371/journal.pone.0102395 (PMC4103819; doi:10.1371/journal.pone.0102395)

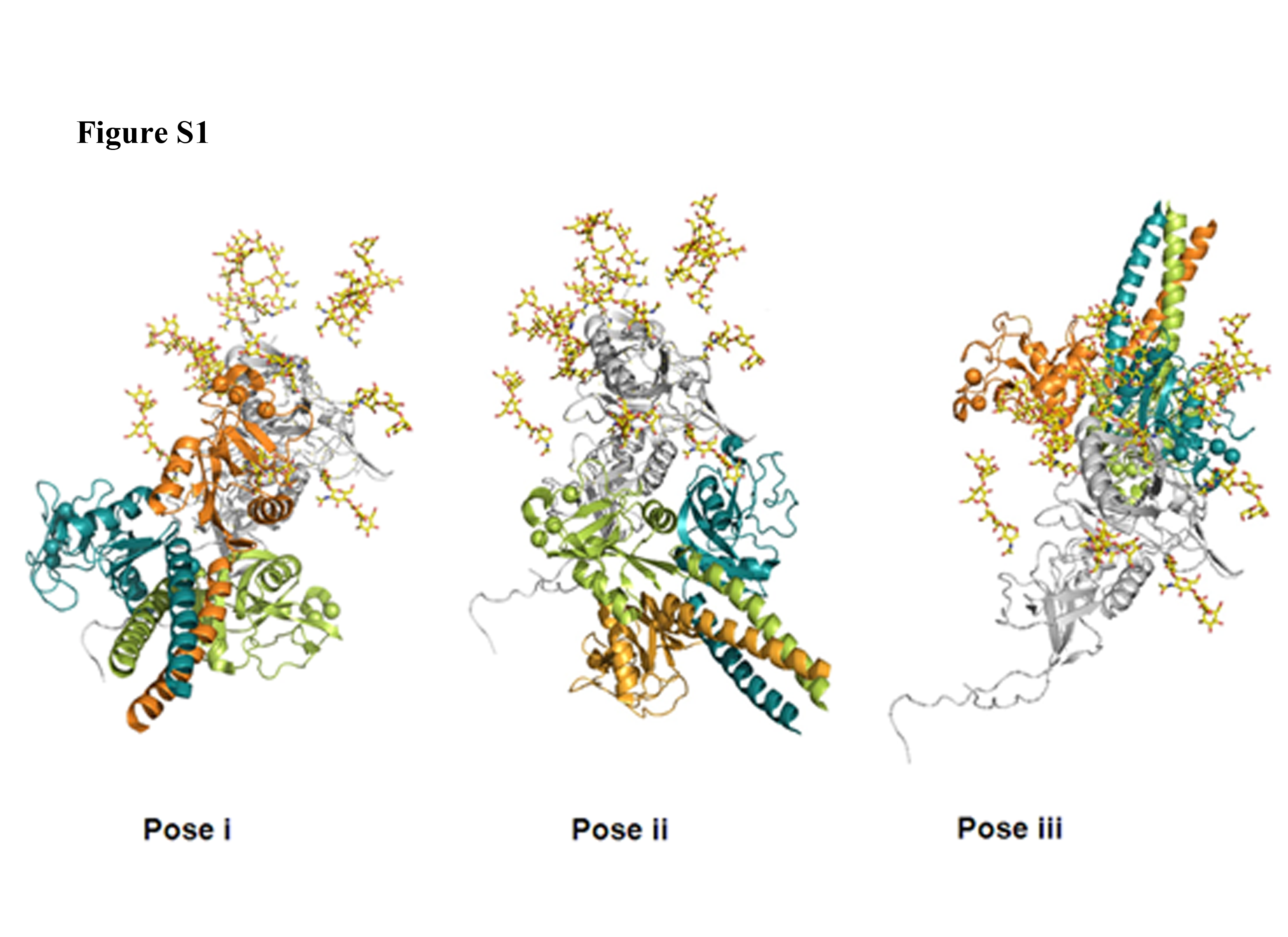

Supplement: Figure S1 — Selected Docked Poses. Patchdock poses (i), (ii) and (iii) exhibiting the CRD of SP-D trimer (light green, dark green and orange cartoon) interacting via glycans modeled on glycoprotein gp120 (grey cartoon) were further refined by FireDock. Best Pose (ii) was further analyzed in the study. (TIF) [file pone.0102395.s001.tif]

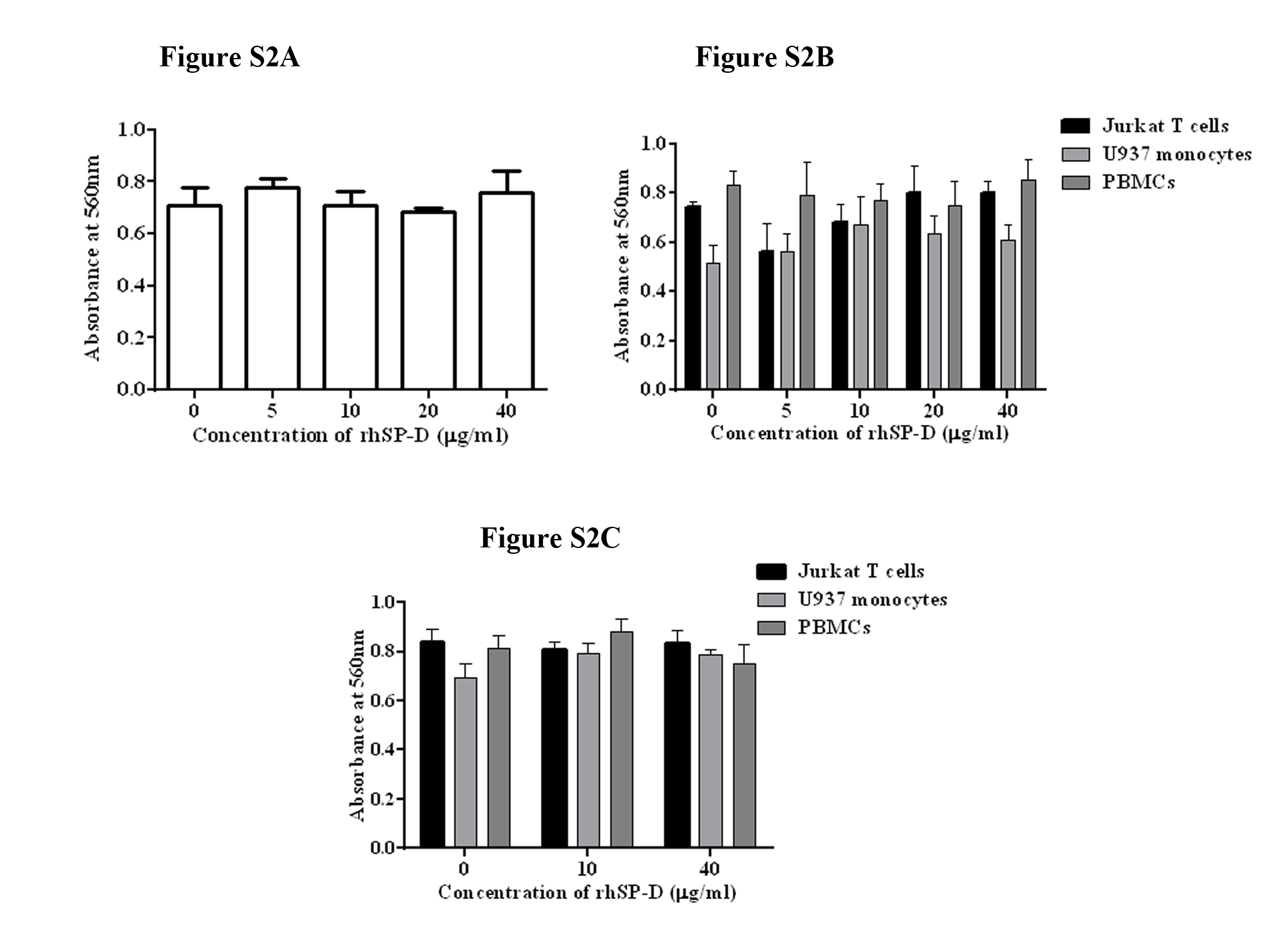

Supplement: Figure S2 — MTT assay to evaluate viability of rhSP-D treated uninfected and infected cells. (A) Viability of TZM-bl cells treated with rhSP-D at 48 h post-infection. Each bar represents the mean ± S.D. (n = 4). (B) Viability of Jurkat T cells, U937 monocytes and activated PBMCs treated with rhSP-D on day 12 of infection. Each bar represents the mean ± S.D. (n = 3). (C) Viability of Jurkat T cells, U937 monocytes and activated PBMCs treated with rhSP-D at 24 h post HIV-1 infection. Each bar represents the mean ± S.D. (n = 3). These different time points with different cells are in coherence with the anti-HIV and differential cytokine expression assays carried out in the present study. Data suggest that viability of cells was not affected by rhSP-D during experiments for anti-HIV activity and differential cytokine expression. (TIF) [file pone.0102395.s002.tif]
