# Supplementary material for: Surfactant Protein D Inhibits HIV-1 Infection of Target Cells via Interference with gp120-CD4 Interaction and Modulates Pro-Inflammatory Cytokine Production
Source: PLoS One. 2014 Jul 18;9(7):e102395. doi: 10.1371/journal.pone.0102395 (PMC4103819; doi:10.1371/journal.pone.0102395)
Supplement: Table S1 — Ranking and energy for solutions refined using FireDock. (DOCX) [file pone.0102395.s003.docx]

**Table S1.**

| **Sr. no.** | **Solution No.** | **Rank** | **Global Energy (Kcal/mol)** |
| --- | --- | --- | --- |
| i | 38 | 27 | 11.95 |
| ii | 39 (Best Pose) | 5 | -20.12 |
| iii | 41 | 44 | 1727.39 |
