# Supplementary material for: Surfactant Protein D Inhibits HIV-1 Infection of Target Cells via Interference with gp120-CD4 Interaction and Modulates Pro-Inflammatory Cytokine Production
Source: PLoS One. 2014 Jul 18;9(7):e102395. doi: 10.1371/journal.pone.0102395 (PMC4103819; doi:10.1371/journal.pone.0102395)
Supplement: Table S2 — Levels of cytokines (pg/ml) in culture supernatants of Jurkat T cells on treatment with indicated concentration of rhSP-D, HIV-1 and HIV-1 and rhSP-D. (DOCX) [file pone.0102395.s004.docx]

**Table S2.**

| **Jurkat T cells:-**  **24 h** |  | **Levels of cytokines (pg/ml) (10^5^cells/ml)** | | | | | | | | | | | | |
| --- | --- | --- | --- | --- | --- | --- | --- | --- | --- | --- | --- | --- | --- | --- |
|  |  | **IL-2** | **IL-4** | **IL-6** | **IL-8** | **IL-10** |  | **VEGF** | **IFN-γ** | **TNF-α** | **IL-1α** | **IL-1β** | **MCP-1** | **EGF** |
| **Cells alone** |  | <0 | 4.09±1.87 | 0.39±0.2 | <0 | <0 |  | <0 | <0 | <0 | <0 | 0.43±0.09 | 12.38±4.32 | <0 |
| **rhSP-D 10µg** |  | <0 | 5.6±2.67 | <0 | 1.83±0.3 | <0 |  | 3.89±1.35 | 1.22±0.54 | <0 | 0.8±0.13 | <0 | 0.58±0.21 | <0 |
| **rhSP-D 40μg** |  | <0 | 6.1±3.2 | <0 | 2.1±0.09 | <0 |  | 5.01±1.98 | <0 | <0 | 1.4±0.5 | <0 | <0 | <0 |
| **HIV** |  | 41.8±12.6 | 4.11±1.18 | 23.75±6.87 | 1346.84±87.34 | 1.6±0.9 |  | 30.96±12.87 | 48.2±3.56 | 32.19±6.98 | 24.59±5.73 | 31.72±6.76 | 16.9±4.21 | <0 |
| **rhSP-D 10µg+HIV** |  | 28.25±7.98 | 4.85±2.56 | 23.48±8.8 | 1416±68.21 | 1.13±1.03 |  | 12.44±2.87 | 28.14±7.93 | 23.7±3.07 | 14.35±3.33 | 26.51±7.89 | 16.13±3.1 | <0 |
| **rhSP-D 40μg+HIV** |  | 18.3±8.2 | 5.89±0.98 | 19.37±3.43 | 1379.9±122.78 | 2.04±0.45 |  | 5.21±1.76 | 13.44±3.45 | 18.07±4.86 | 6.85±2.94 | 31.09±4.23 | 12.9±4.56 | 2.12±1.3 |
