# Supplementary material for: Surfactant Protein D Inhibits HIV-1 Infection of Target Cells via Interference with gp120-CD4 Interaction and Modulates Pro-Inflammatory Cytokine Production
Source: PLoS One. 2014 Jul 18;9(7):e102395. doi: 10.1371/journal.pone.0102395 (PMC4103819; doi:10.1371/journal.pone.0102395)
Supplement: Table S3 — Levels of cytokines (pg/ml) in culture supernatants of U937 cells on treatment with indicated concentration of rhSP-D, HIV-1 and HIV-1 and rhSP-D. (DOCX) [file pone.0102395.s005.docx]

**Table S3.**

| **U937 monocytes cells:- 24 h** |  | **Levels of cytokines (pg/ml) (10^5^cells/ml)** | | | | | | | | | | | |
| --- | --- | --- | --- | --- | --- | --- | --- | --- | --- | --- | --- | --- | --- |
|  |  | **IL-2** | **IL-4** | **IL-6** | **IL-8** | **IL-10** | **VEGF** | **IFN-γ** | **TNF-α** | **IL-1α** | **IL-1β** | **MCP-1** | **EGF** |
| **Cells alone** |  | <0 | 4.61±1.32 | <0 | 7.58±2.54 | <0 | 70.25±21.56 | <0 | <0 | <0 | 0.47±0.14 | 45.44±12.72 | <0 |
| **rhSP-D 10µg** |  | 1.09±0.25 | 5.57±2.83 | 0.37±0.12 | 9.85±4.45 | <0 | 80.6±5.85 | 1.29±0.76 | 1.54±0.43 | 1±0.34 | 0.98±0.23 | 34.27±7.21 | <0 |
| **rhSP-D 40μg** |  | <0 | 10.9±3.6 | 1.7±0.56 | 23.9±6.87 | <0 | 78.9±9.94 | 3.98±3.2 | <0 | <0 | <0 | 48.7±9.71 | <0 |
| **HIV** |  | 237.4±32.95 | 5.89±2.80 | 81.41±8.77 | >1646 | 1.23±0.52 | 220.4±12.76 | 38.55±6.21 | 4.2±3 | 1.35±0.87 | 53.74±12.63 | 789.61±59.54 | 1.05±0.52 |
| **rhSP-D 10µg+HIV** |  | 151.38±21.87 | 5.73±1.11 | 50.33±10.83 | >1646 | <0 | 127.97±27.66 | 18.65±2.56 | 4.53±2.34 | 1.23±0.53 | 44.03±6.84 | 429.5±34.41 | <0 |
| **rhSP-D 40μg+HIV** |  | 53.336±8.08 | 5.73±0.76 | 14.51±4.73 | >1646 | 6.21±2.74 | 65.7±6.04 | 4.96±1.41 | 8.45±3.88 | 0.92±0.14 | 38.56±2.65 | 73.43±18.92 | <0 |
