# Supplementary material for: Surfactant Protein D Inhibits HIV-1 Infection of Target Cells via Interference with gp120-CD4 Interaction and Modulates Pro-Inflammatory Cytokine Production
Source: PLoS One. 2014 Jul 18;9(7):e102395. doi: 10.1371/journal.pone.0102395 (PMC4103819; doi:10.1371/journal.pone.0102395)
Supplement: Table S4 — Levels of cytokines (pg/ml) in culture supernatants of PBMCs on treatment with indicated concentration of rhSP-D, HIV-1 and HIV-1 and rhSP-D. (DOCX) [file pone.0102395.s006.docx]

**Table S4.**

| **PHA activated PBMCs:- 24 h** |  | **Levels of cytokines (pg/ml) (10^5^cells/ml)** | | | | | | | | | | | |
| --- | --- | --- | --- | --- | --- | --- | --- | --- | --- | --- | --- | --- | --- |
|  |  | **IL-2** | **IL-4** | **IL-6** | **IL-8** | **IL-10** | **VEGF** | **IFN-γ** | **TNF-α** | **IL-1α** | **IL-1α** | **MCP-1** | **EGF** |
| **Cells alone** |  | 44.68±8.41 | 12.14±3.72 | 69.53±7.52 | 183.67±21.7 | 8.19±1.81 | 15.01±3.21 | 11.03±2.17 | 23.13±3.76 | 8.67±3.76 | 10.39±2.31 | 758.06±36.91 | 1.95±0.81 |
| **rhSP-D 10µg** |  | 21.72±7.77 | 13.81±4.21 | 58.13±6.17 | 163.14±12.9 | 6.23±2.81 | 9.83±2.1 | 7.32±1.8 | 11.54±1.73 | 6.31±2.17 | 12.21±1.81 | 651.86±63.78 | 1.89±0.26 |
| **rhSP-D 40μg** |  | 11.5±7.96 | 15.13±2.36 | 49.16±10.29 | 112.65±32.98 | 6.58±1.15 | 3.32±0.73 | 5.42±2.34 | 6.43±1.87 | 6.18±3.14 | 9.86±3.14 | 613.72±49.71 | 2.16±0.47 |
| **HIV** |  | 69.41±8.98 | 9.73±4.43 | 125.55±23.61 | >1646 | 8.89±3.64 | 31.93±6.17 | 26.98±7.36 | 42.22±8.76 | 25.25±3.61 | 36.42±5.38 | 853.63±18.96 | 2.22±0.38 |
| **rhSP-D 10µg+HIV** |  | 51.07±4.13 | 12.46±3.32 | 106.63±12.85 | >1646 | 10.63±4.2 | 19.95±2.65 | 19.73±1.64 | 25.32±1.89 | 18.68±2.54 | 23.18±1.54 | 795.87±43.75 | 0.91±0.16 |
| **rhSP-D 40μg+HIV** |  | 18.71±1.47 | 12.63±6.3 | 78.93±32.78 | >1646 | 9.96±3.6 | 5.61±0.65 | 7.09±1.56 | 8.08±1.54 | 10.01±1.55 | 9.76±2.71 | 754.98±65.98 | 1.18±0.81 |
